# Evaluating the Immunogenicity and Protective Efficacy of a Novel Vaccine Candidate Against *Salmonella* in Poultry

**DOI:** 10.3390/vaccines14010068

**Published:** 2026-01-07

**Authors:** Roshen N. Neelawala, Varsha Bommineni, Chaitanya Gottapu, Lekshmi K. Edison, Krishni K. Gunathilaka, Gary D. Butcher, John F. Roberts, Subhashinie Kariyawasam

**Affiliations:** 1 Department of Comparative, Diagnostic, and Population Medicine, College of Veterinary Medicine, University of Florida, Gainesville, FL 32608, USA; roshen.neelawala@ufl.edu (R.N.N.); edison.le@ufl.edu (L.K.E.);; 2 Department of Large Animal Clinical Sciences, College of Veterinary Medicine, University of Florida, Gainesville, FL 32608, USA; 3 Bronson Animal Disease Diagnostic Laboratory, Florida Department of Agriculture and Consumer Services, Kissimmee, FL 34741, USA

**Keywords:** chickens, immunogenicity, InvG, non-typhoidal *Salmonella* (NTS), poultry, subunit vaccine, vaccination

## Abstract

**Background**: Non-typhoidal *Salmonella* (NTS) is a major foodborne pathogen, with poultry products, especially eggs, being the primary source of human infections. Current serovar-specific poultry vaccines effectively reduce targeted *Salmonella* serovars but may inadvertently promote the emergence of untargeted serovars within poultry flocks. Therefore, novel vaccine candidates providing broad cross-serovar protection are needed to improve overall effectiveness of *Salmonella* control programs. **Objectives**: This study evaluated the immunogenicity of the novel subunit vaccine candidate InvG and assessed its ability to reduce *Salmonella* colonization in vaccinated laying hens and their progeny through maternally derived antibodies transferred via egg yolk. **Methodology**: Three experiments were performed. Experiment I evaluated the immunogenicity of purified recombinant InvG by (a) measuring anti-InvG antibodies using an enzyme-linked immunosorbent assay (ELISA) and (b) completing transcriptomic profiling of immune responses in vaccinated chickens. Vaccinated chickens were subsequently challenged with *Salmonella* Enteritidis to assess the efficacy of anti InvG antibodies in reducing intestinal colonization of *Salmonella*. Experiment II involved immunizing hens with InvG, to evaluate passive transfer of antibodies via egg yolk and the protective efficacy of maternally derived antibodies against *Salmonella* challenge. Passive transfer was assessed by measuring IgY antibodies in hen serum, egg yolk, and progeny serum, as well as secretory IgA (sIgA) antibodies in progeny intestinal washings using ELISA. Protective efficacy was evaluated by orally challenging one-day-old chicks with three different *Salmonella* serovars. Experiment III assessed the persistence of anti-InvG antibodies in the serum of vaccinated hens and their transfer into eggs following two doses of InvG. **Results**: InvG vaccination induced robust IgY antibody responses in hens, with efficient maternal antibody transfer to progeny via egg yolk. A statistically significant reduction in *Salmonella* colonization was observed in both vaccinated hens and their progeny. **Conclusions**: These findings demonstrate that InvG represents a promising subunit vaccine candidate for *Salmonella* control in poultry and warrants further investigation towards development as a broadly protective commercial poultry vaccine against *Salmonella*.

## 1. Introduction

Non-typhoidal *Salmonella* (NTS) is a major foodborne pathogen that poses a significant public health risk, with poultry products consistently identified as the primary source of human infections [1,2]. Due to the significant impact of foodborne salmonellosis on public health, the poultry industry faces increasing pressure to control *Salmonella* colonization in flocks, which helps reduce *Salmonella* contamination of poultry products intended for human consumption. Currently, vaccination, stringent biosecurity practices, and screening of poultry flocks for the presence of *Salmonella* are used as primary tools to reduce *Salmonella* colonization in poultry, particularly in commercial layer flocks [2]. Once infected, poultry can harbor *Salmonella* asymptomatically throughout their entire lifespan, with intermittent shedding of these bacteria, contaminating poultry products and the farm environment [3]. Of particular concern are *S.* Enteritidis (serogroup D) and *S*. Typhimurium (serogroup B), which are recognized as the predominant serovars associated with foodborne salmonellosis in humans linked to poultry [4,5,6]. Due to the public health risks posed by *S*. Enteritidis and *S*. Typhimurium, the poultry industry has faced significant regulatory pressure to implement control measures for these serovars. Over the past decades, the widespread implementation of vaccination programs, utilizing both live attenuated and inactivated *Salmonella* vaccines, has substantially reduced the prevalence of these serovars in poultry flocks [7,8,9,10,11,12]. However, current vaccines exhibit limited efficacy in providing complete protection, and their efficacy is primarily serovar-specific [2,13]. Thus, the serovar-specific approach presents a significant limitation, as it unintentionally grants a competitive advantage for non-targeted serovars to proliferate without restraint. For example, serogroup C *Salmonella* serovars, such as *S*. Infantis and *S*. Kentucky [14,15] have become more prevalent in poultry over the last decade, as they fall outside of the serovars targeted by the currently available vaccines [13]. These untargeted serovars pose a considerable challenge to the poultry industry. They hinder efforts to control overall *Salmonella* contamination of poultry products and increase public health risk, due to their acquisition of multidrug resistance (MDR) [15]. To address the emergence of these serovars, it is crucial to identify new vaccine candidates that offer broader protection against multiple serovars, thereby preventing their spread and enhancing overall *Salmonella* control. Research has demonstrated that vaccines containing conserved antigens, such as some outer membrane proteins (OMPs), can induce cross-protection against heterologous serovars, thereby enhancing the overall efficacy of *Salmonella* control strategies in poultry [16,17]. Another significant challenge in *Salmonella* control is the early exposure and colonization of chicks with *Salmonella*, either *in-ovo* or immediately after hatching. Newly hatched chicks, driven by innate foraging behavior and exposure to fluff from infected birds in the hatcher, are particularly vulnerable to *Salmonella* [18]. This early encounter with *Salmonella* occurs while the chick’s immune system is still underdeveloped, and the chick relies heavily on maternal antibodies for protection. The gut-specific immune system of chicks is shown to be developed around two weeks post-hatch [19,20]. This passive protection is crucial in protecting the chick during the vulnerable period, before its adaptive immune system reaches functional maturity [21]. Chicks acquiring *Salmonella* during early developmental stages, such as in the hatchery environment, can potentially serve as a source of widespread dissemination of the pathogen. This early colonization can lead to extensive dissemination of *Salmonella* among flock mates, contaminating the farm environment and poultry products destined for the market [22]. It has also been demonstrated that primary exposure to *Salmonella* during early developmental stages leads to more prolonged colonization than initial exposure in mature chickens [23].

Both controlling *Salmonella* in early life and understanding the pivotal role of maternally derived antibodies in preventing early-life infections are of critical importance. As a result, assessing the immune response in vaccinated hens and the subsequent transfer of these antibodies to progeny via eggs provides crucial information when evaluating novel vaccine candidates. In this study, we evaluated a novel vaccine candidate, InvG, which is a highly conserved structural component of the type 3 secretion system (T3SS), present in various pathogenic Gram-negative bacteria, including *Salmonella*. This study was conducted with three primary objectives: (1) to assess the immunogenicity of InvG as a vaccine candidate antigen, (2) to assess the effectiveness of InvG in reducing *Salmonella* colonization in vaccinated hens, and (3) to evaluate the transfer of anti-InvG antibodies from vaccinated hens to their progeny and the protective effect of these maternally derived antibodies on reducing *Salmonella* colonization in progeny chicks.

## 2. Materials and Methods

### 2.1. Bacterial Strains, Plasmids, and Media

Bacteria used for challenge experiments included three serovars of *Salmonella* isolated from commercial layer chickens: *S*. Enteritidis, *S*. Heidelberg, and *S*. Kentucky. *S*. Enteritidis (NZ_CP011790) and *S*. Heidelberg were isolated from shell eggs, whereas *S.* Kentucky was isolated from the yolk sac of a one-day-old broiler chick. *Escherichia coli* M15 strain (QIAGEN, Germantown, MD, USA) was used as the expression host and pQE-30 expression vector (QIAGEN) was used as the cloning vector for recombinant InvG expression. All bacterial strains were cultured in Luria-Bertani (LB) broth without antibiotic supplementation unless otherwise specified. Quantitative *Salmonella* enumeration was conducted on xylose lysine tergitol-4 (XLT-4) selective agar medium (Thermo Fisher Scientific, Waltham, MA, USA).

### 2.2. Determination of InvG Conservation Across NTS Serovars

Protein sequences were obtained from the UniProt database [24] using the search terms “InvG” and the specific serovar of interest. A total of 10 sequences were selected from *S.* Enteritidis (serogroup D1) and *S.* Typhimurium (serogroup B), while the remaining serovars were represented by all available sequences: 9 from *S.* Dublin (serogroup D1), 5 from *S.* Infantis (serogroup C1), and 3 from *S.* Heidelberg (serogroup B). These sequences were compiled into a master sequence file and subjected to multiple sequence alignment using the Clustal Omega multiple sequence alignment tool [25] (https://www.ebi.ac.uk/jdispatcher/msa/clustalo?stype=protein, accessed on 20 January 2022). The resulting multiple sequence alignment was analyzed using the ConSurf online tool to determine the conservation status of *invG* across common NTS serovars [26]. *S*. Enteritidis sequence (UniProt ID: A0A0W3PV57) was selected as the reference sequence for this study.

### 2.3. Cloning and Expression of InvG in Escherichia coli

**Figure 1:**
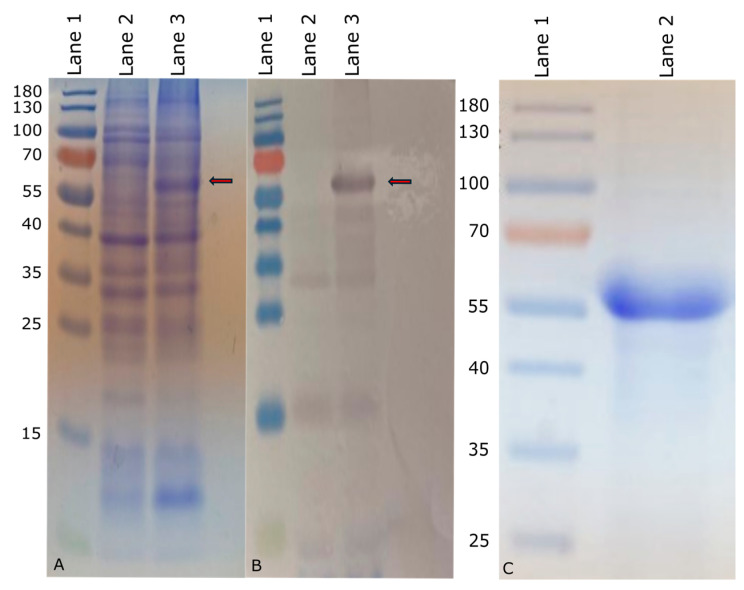
Expression of InvG in M15 *E. coli* (marked with red arrow). (**A**) Coomassie-stained SDS-PAGE of expressed InvG. (**B**) Western blot of expressed InvG. Lane1: PageRuler™ prestained protein ladder (Thermo Fisher Scientific) marked in kDa; Lane 2: Uninduced bacterial culture (negative control); Lane 3: Bacterial culture induced with IPTG. (**C**) Purified InvG on Coomassie-stained SDS-PAGE. Lane 1: PageRuler™ prestained protein ladder (Thermo Fisher Scientific) marked in kDa; Lane 2: Nickel-nitrilotriacetic acid (Ni-NTA)-purified InvG.

The nucleotide sequence corresponding to *invG* was PCR amplified from the *S.* Enteritidis SEE1 genome (GenBank Accession: NZ_CP011790) using a pair of gene-specific primers that contained *Bam*HI and *Kpn*I restriction sites at 5′ or 3′ ends, respectively (underlined). The forward primer was CGCGCGGATCCAAGACACATATTCTTTTGGCCAGAGTGCTG, and the reverse primer was GCCGCGGTACCTCATTTAATTGCCTCCTGACCTCTATCCAGA. The PCR was performed with SEE1 genomic DNA as the template to amplify the full-length *invG*. The PCR product was subjected to gel electrophoresis on a 1% agarose gel and the amplicon was purified using the MiniElute Gel Extraction Kit (QIAGEN) and digested with *Bam*HI and *Kpn*I enzymes (New England Biolabs, Ipswich, MA, USA). The product was subsequently ligated into the pQE-30 expression vector (QIAGEN) using T4 ligase (New England Biolabs). The resulting plasmid was introduced into the *E. coli* host strain M15 (QIAGEN), which contained the pREP4 plasmid conferring kanamycin resistance and expressing *lac* repressor (*lacI*) constitutively. The accuracy of the cloned *invG* sequence was verified by sequencing the cloning junctions of the recombinant plasmid. The verified clone was used for large batch expressions of InvG. To induce recombinant protein expression, isopropyl β-D-1-thiogalactopyranoside (IPTG) (Thermo Fisher Scientific) was added at a final concentration of 1 mM. The expression of 6× His-tagged InvG recombinant protein was verified through a combination of colony blot analysis according to the manufacturer’s instructions [27], which utilized anti-poly-histidine antibodies (QIAGEN), followed by sodium dodecyl sulfate-polyacrylamide gel electrophoresis (SDS-PAGE) to identify high-expression clones (Figure 1A,B).

### 2.4. Purification of InvG

The recombinant InvG protein with an approximate molecular mass of 63 kDa was subsequently purified by passing through a nickel-nitrilotriacetic acid (Ni-NTA) agarose column (QIAGEN). The eluate containing the purified recombinant protein was fractionated by SDS-PAGE (Figure 1C). The confirmation of protein identity and purity was achieved through Western blot analysis using RGS-His antibodies (QIAGEN). The fractions containing the recombinant protein were concentrated using Amicon (Sigma-Aldrich, Inc., St. Louis, MO, USA) ultrafiltration devices with a 30 kDa cutoff, followed by quantitative protein determination using a Pierce BSA protein assay (Thermo Fisher Scientific) or Qubit™ Protein Assay Kit (Thermo Fisher Scientific). Purified InvG was analyzed for lipopolysaccharide (LPS) contamination by silver staining, following SDS-PAGE, to confirm that the purified protein preparation was devoid of LPS.

### 2.5. Chickens

Chickens were either hatched from fertile specific pathogen-free (SPF) White Leghorn chicken eggs obtained from AVSBio (Charleston, SC, USA) in-house or purchased as adult hens or cockerels from a commercial farm known to be free of *Salmonella*. Before the experiments, cloacal swabs taken from all chickens were tested for *Salmonella* by culture to confirm their *Salmonella*-free status. Chickens were either housed in isolators or on the floor with litter shavings in isolation units, depending on the age of the chickens and the nature of the experiment. All chickens had free access to water and age-appropriate feed rations. When embryonated eggs were required, hens were provided with increased light (14 h/day) and specialized layer feed on a gradually incremented schedule to optimize laying, while minimizing stress on chickens. Experimental procedures and animal care were performed in compliance with applicable federal and institutional animal care policies and guidelines. The experimental protocol for handling animals was approved by the Institutional Animal Care and Use Committee at the University of Florida (Protocol Numbers: 202300000617 and 202011243). Chickens were housed with a minimum of 2 square feet per chicken. Chickens exhibiting signs of distress or disease were humanely euthanized. All chickens were euthanized using the American Veterinary Medical Association (AVMA) approved methods.

### 2.6. Sample Collection

Blood was collected from the wing vein of adult hens and by cardiac puncture immediately after euthanasia in one-day-old chicks. The liver and spleen samples, along with cecal contents, were collected in 2 mL microcentrifuge tubes containing 2.8 mm stainless steel beads, following aseptic techniques. Intestinal washings were collected from two distinct anatomical regions, depending on the age of the chicken. In adult hens, samples were obtained from the proximal 30 cm of the small intestine, beginning at the duodenum. In one-day-old chicks, washings were collected from a 14 cm section centered on the Meckel’s diverticulum (7 cm on each side). To collect the washings, the intestinal segments were lavaged with sterile phosphate-buffered saline (PBS), immediately placed on ice, and subsequently stored at −20 °C until analysis.

Eggs were collected daily from the pen and stored at 4 °C, prior to processing for the measurement of maternal antibody titers. Under aseptic conditions, the egg yolk was carefully aspirated using a sterile syringe and needle, then transferred into a separate sterile container. Egg yolks from two eggs were pooled and homogenized to be treated as a single sample for ELISA analysis.

### 2.7. Measurement of Antibodies in Chicken Sera, Egg Yolk, and Intestinal Washings

Indirect ELISA was employed to detect serum and intestinal mucosal antibody titers against InvG. Serum and intestinal washings were diluted in PBS and tested in duplicates. All incubation steps were carried out at room temperature for 2 h. For the ELISA, 96-well polystyrene plates (Immulon, Waltham, MA, USA) were coated with purified recombinant InvG (50 μL of 100 µg/mL solution in PBS) overnight at 4 °C. After coating, the plates were washed three times with Tris-buffered saline containing 0.05% Tween-20 (TBST), prior to blocking with 300 μL of 3% bovine serum albumin for 2 h at room temperature. Primary antibody incubation was performed using 50 μL of diluted samples: hyperimmune serum (1:1000 dilution), intestinal washings (1:50 dilution), or egg yolk (1:1000 dilution). The plates were washed with TBST three times, followed by the addition of 200 μL of the secondary antibody diluted 1:2000 to each well, and incubated at room temperature for 2 h. Alkaline phosphatase-conjugated rabbit anti-chicken antibodies (Abnova, Neihu District, Taipei City, Taiwan) and alkaline phosphatase-conjugated goat anti-chicken IgA heavy chain secondary antibody (Novus Biologicals, Centennial, CO, USA) were used as secondary antibodies for the detection of serum IgY (IgG) and secretory IgA (sIgA), respectively. After another wash step, 100 μL of *p*-nitrophenyl phosphate substrate (Thermo Fisher Scientific) was added and incubated for 15 min for color development. The reaction was stopped by adding 2 M NaOH, and optical density (OD) readings were taken at 405 nm wavelength on a Biotek 800 TS microplate reader (Agilent Technologies, Santa Clara, CA, USA). Optical density readings were adjusted for the blank readings, and the ELISA readings were normalized with positive controls that were run on each plate, to account for plate-to-plate variability. The final titers are expressed as a proportion of the positive control (final titer = observed OD value of sample/OD value of positive control).

### 2.8. Chicken Vaccination and Salmonella Challenge Procedures

Chickens were vaccinated with 50 µg of purified InvG, combined with 50 µg of Quil-A adjuvant (InvivoGen, San Diego, CA, USA). The vaccine was administered intramuscularly into the breast muscle using a 23 G needle. For the oral challenge with *Salmonella*, bacteria were grown in LB broth overnight at 37 °C in a shaking incubator. The overnight culture was then diluted to 1:200 in LB and incubated with shaking at 37 °C for another 10 h to harvest bacteria in the log phase of the growth curve (approximately 10^7^ bacteria/chicken). Bacterial numbers in the inocula were verified the next day by calculating the colony-forming units (CFUs) in serial dilutions.

### 2.9. RNA Extraction and rRNA Depletion

Tissue samples from the liver, spleen, ovary, and cecal wall were collected in RNAlater (Thermo Fisher Scientific), a week after the second vaccination, and stored at −80 °C until analysis. Total RNA extraction was performed using the RiboPure RNA Purification Kit (Invitrogen, Waltham, MA, USA), following the manufacturer’s instructions. Ribosomal RNA (rRNA) was depleted using the RiboMinus™ Eukaryote System v2 (Invitrogen). The quality and quantity of RNA were assessed using a NanoDrop 2000/2000c spectrophotometer (Thermo Fisher Scientific) and Qubit™ RNA High Sensitivity Assay Kit (Invitrogen) at each step of the procedure.

### 2.10. Library Preparation and RNA Sequencing

RNA libraries were constructed using the TruSeq Stranded mRNA Library Kit (Illumina Inc., San Diego, CA, USA), following the manufacturer’s protocol. First, the depleted RNA was enzymatically fragmented, and then reverse transcribed into first-strand cDNA, using SuperScript II Reverse Transcriptase (Invitrogen) with random hexamer primers. Second-strand cDNA synthesis was performed using DNA polymerase, and dUTP was incorporated in place of dTTP to achieve a marked second strand that was subsequently quenched. cDNA purification was performed using AMPure XP magnetic beads (Beckman Coulter, Brea, CA, USA). The purified cDNA underwent end repair to generate blunt-ended fragments. This was followed by 3’ adenylation, which added a single adenine nucleotide to the 3’ ends of the blunt fragments, to facilitate efficient adapter ligation while preventing self-ligation. TruSeq RNA Single Indexes (Illumina Inc.) were ligated to the A-tailed cDNA fragments as sequencing adapters. Libraries were enriched through limited-cycle PCR amplification, and a final round of library cleanup was performed using AMPure XP beads (Beckman Coulter). The quality and size distribution of the libraries were verified using an Agilent TapeStation system (Agilent Technologies). The final libraries were normalized, pooled, and sequenced on a NovaSeq X Series platform (Illumina Inc.).

### 2.11. RNA Sequence Analysis

The output sequencing reads were analyzed using the CLC Genomics Workbench version 25.0.3 (QIAGEN). Adapter sequences and low-quality bases (Phred score < 30) were trimmed from the sequencing data to maintain a base call accuracy of more than 99.9%. The remaining high-quality reads were mapped and aligned to the *Gallus gallus* White Leghorn reference genome assembly (GCF_016700215.2) from the National Center for Biotechnology Information (NCBI) repository. Three replicates per organ in each group (vaccinated vs. unvaccinated) were used. TMM normalization (Trimmed Mean of M values) was used to normalize the gene expression data. Differential gene expression (DGE) between vaccinated and unvaccinated groups was evaluated using the CLC Genomics Expression Browser tool, which utilizes a modified statistical analysis package based on the edgeR Bioconductor package by Robinson et al., 2009 [28]. The DEG data were then filtered using the following parameters: false discovery rate (FDR) ≤ 0.05, (FC) ≥ 2.0, and *p*-value ≤ 0.01. Gene ontology (GO) analysis was conducted using CLC Genomics Workbench and NCBI. Gene enrichment analysis was conducted using the web-based tool iDEP version 2.2 [29].

### 2.12. Enumeration of Salmonella

Each sample was weighed individually, and an equivalent volume (*w*/*v*) of buffered-peptone water (BPW) (Thermo Fisher Scientific) was added at a ratio of 1 μL BPW per 1 mg tissue, ensuring that 100 μL of homogenate represented 50 mg of tissue. Typical tissue masses were 0.7–0.8 g for cecal samples, 0.5 g for the liver, and 0.5 g for the spleen. Samples were homogenized using a BioSpec Mini-BeadBeater (BioSpec Products Inc., Bartlesville, OK, USA) for 20 s. Following homogenization, the samples underwent serial dilutions in PBS and were subsequently plated on XLT-4 agar (Thermo Fisher Scientific) for quantification of *Salmonella*. The plates were incubated for 48 h, and the characteristic *Salmonella* colonies—identified by their black centers and pink margins—were counted to calculate the number of *Salmonella* per gram of tissue. *Salmonella* counts were expressed as colony-forming units (CFUs) per gram of the tissue.

### 2.13. Experimental Design

Three chicken experiments were conducted in this study. Experiment I evaluated the immunogenicity and efficacy of InvG administered via the intramuscular route for protecting chickens against a *Salmonella* challenge. Experiment II assessed the effectiveness of maternally transferred anti-InvG antibodies in protecting progeny chicks hatched from eggs of vaccinated chickens following a *Salmonella* challenge. Experiment III was designed to evaluate the persistence of anti-InvG antibodies in the serum of vaccinated hens and their transfer into eggs following two doses of InvG, as excessive intramuscular vaccination may adversely affect flock uniformity and increase production costs.

In Experiment I, 78 one-day-old chicks hatched from SPF eggs were randomly allocated into three groups. Group 1 (*n* = 29) received three vaccinations of 50 µg of InvG mixed with 50 µg of QuilA adjuvant at the ages of two, four, and six weeks, intramuscularly. Chickens in Group 2 (*n* = 29) and 3 (*n* = 20) received a similar volume of PBS intramuscularly. One week after the last vaccination, anti-InvG IgY titers in all three groups were measured using ELISA, and then groups 1 and 2 were challenged with 10^10^ CFU of *S.* Enteritidis strain SEE1 in 0.5 mL of PBS via oral gavage, while group 3 received 0.5 mL of PBS only. Prior to challenge, 9 chickens each from groups 1 and 2 were euthanized for tissue collection and RNA extraction for host transcriptome analyses. Blood samples were collected from each chicken before the challenge and at the time of euthanasia, to determine the serum IgY antibody response in the vaccinated group, compared to that in the unvaccinated group. Intestinal washings were obtained exclusively during post-mortem examination, as this procedure required euthanasia. The experiment was repeated to verify the results.

In Experiment II, twenty hens in peak production were randomly allocated into two groups: treatment (*n* = 13) and control (*n* = 7). These chickens were obtained as day-old chicks from a commercial hatchery, without *Salmonella* vaccination and testing negative for *Salmonella*. Hens were kept with a cockerel per group to obtain fertile eggs. The treatment group received three doses of InvG + QuilA in 100 µL of PBS intramuscularly at 33, 35, and 37 weeks of age, while the control group received equivalent volumes of PBS. Serum samples were collected biweekly after each vaccination to compare anti-InvG IgY titers between vaccinated and unvaccinated groups using ELISA. Egg collection commenced following the second vaccination and continued at weekly intervals for five weeks. The collected eggs were either used to measure egg yolk IgY (*n* = 5) or incubated to obtain one-day-old chicks on a weekly basis. From each hatch, a subset of one-day-old chicks (*n* = 5) was used to determine intestinal sIgA, while another subset (*n* = 5) was challenged orally with 10^7^ CFU of one of three *Salmonella enterica* serovars: *S*. Enteritidis, *S*. Typhimurium, or *S*. Kentucky. Vaccine efficacy was evaluated by comparing *Salmonella* counts in the cecum and visceral organs (liver and spleen) between the vaccinated challenged group and the unvaccinated challenged group. The unvaccinated and unchallenged groups served as negative controls. At the end of the egg collection period, the adult hens were euthanized to collect intestinal washings for sIgA evaluation.

In Experiment III, chicks were obtained by hatching SPF eggs and were randomly allocated into two groups (*n* = 4): vaccinated and unvaccinated. These chickens were tested for *Salmonella* and the vaccinated group was administered two doses of vaccine preparation (50 µg of InvG mixed with 50 µg of QuilA adjuvant) intramuscularly at 17 and 19 weeks of age. The unvaccinated group received an equal volume of sterile PBS intramuscularly. Blood and eggs were collected from these hens weekly for a period of 5 weeks to determine their serum IgY and egg yolk antibodies against InvG.

### 2.14. Statistical Analysis

Animal and sample numbers were determined by using the OpenEpi online tool [30]. The power and confidence intervals were set at 80% and 95%, respectively. The data were analyzed to determine significant differences in *Salmonella* colonization levels in the cecal contents and internal organs (liver and spleen), between the vaccinated challenged and unvaccinated challenged chicken groups. As the data were not normally distributed, the Mann–Whitney U test, a non-parametric method, was employed to compare the two groups under the null hypothesis that *Salmonella* levels would be the same in both groups. For the ELISA tests measuring IgY in serum and egg yolk, as well as sIgA in intestinal washings, the Student’s *t*-test was used to evaluate significant differences between the vaccinated and unvaccinated groups. A *p*-value of ≤ 0.05 was considered statistically significant.

## 3. Results

### 3.1. Conservation of InvG Protein Across NTS Serovars

**Figure 2:**
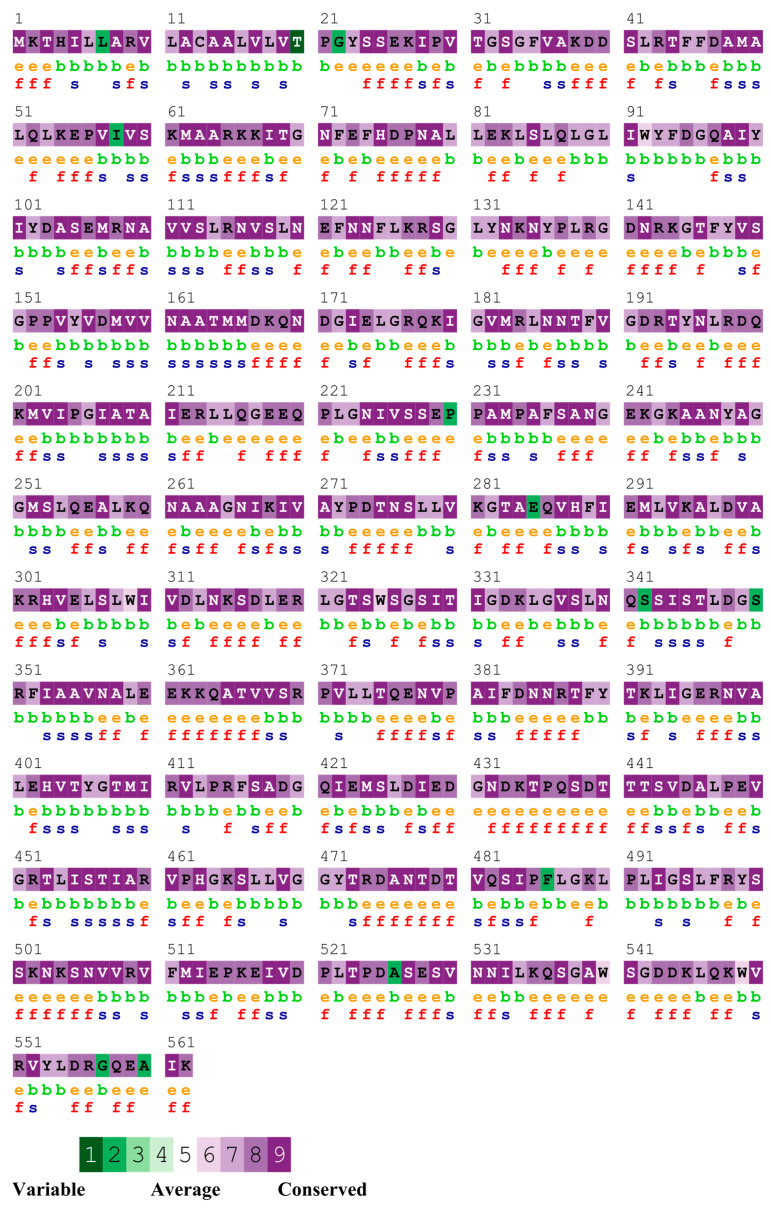
Sequence conservation of InvG across the serovars. e—An exposed residue according to the neural network algorithm; b—a buried residue according to the neural network algorithm; f—a predicted functional residue (highly conserved and exposed); s—a predicted structural residue (highly conserved and buried).

The sequence analysis revealed that InvG exhibited high conservation and more than 99% identity across various serovars at the amino acid level examined in this comparison (Figure 2).

### 3.2. Experiment I—Immunogenicity and Protective Efficacy of Intramuscularly Administered InvG Against Salmonella Challenge in Chickens

#### 3.2.1. Immune Response to InvG Recombinant Protein Vaccination

**Figure 3:**
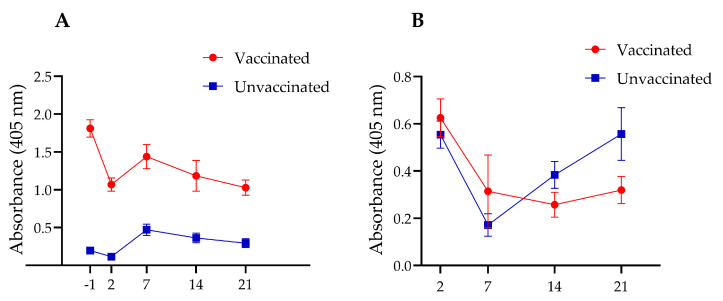
Time-course analysis of IgY levels in the serum (**A**) and sIgA levels in intestinal washings (**B**) of vaccinated and unvaccinated chickens at one day before (−1) and 2, 7, 14, and 21 days after *Salmonella* challenge. InvG was administered via the intramuscular route at two, four and six weeks of age.

In Experiment I, the vaccinated chickens exhibited a robust serum IgY antibody response, compared to the unvaccinated group. Serum collected prior to *Salmonella* challenge showed more than a nine-fold increase in anti-InvG IgY levels in the vaccinated group. At two days post-challenge, IgY antibody levels declined in both vaccinated and unvaccinated groups; however, the levels remained higher in vaccinated chickens than in control chickens. IgY levels increased at 7 days post-challenge before subsequently declining. Despite these fluctuations, throughout the duration of the experiment (i.e., 21 days post-challenge), vaccinated chickens consistently maintained higher IgY levels, compared with their unvaccinated counterparts (Figure 3A).

Although higher intestinal sIgA levels were observed in the vaccinated group during the first two post-mortem time points (2 and 7 days post-challenge), as compared with the unvaccinated group, these differences were not statistically significant (Figure 3B). However, the unvaccinated group exhibited a delayed but significant rebound in sIgA levels at later postmortem timepoints (14 and 21 days post-challenge), with a statistically significant increase observed at 21 days post-challenge. These findings suggest that vaccination may trigger an early mucosal immune response that initially declines as *Salmonella* is cleared from the intestines and subsequently stabilizes over time. In contrast, unvaccinated chickens experience an initial suppression of sIgA levels, followed by a delayed compensatory increase in response to persistent *Salmonella* colonization.

#### 3.2.2. Reduction of Cecal *Salmonella* Colonization

**Figure 4:**
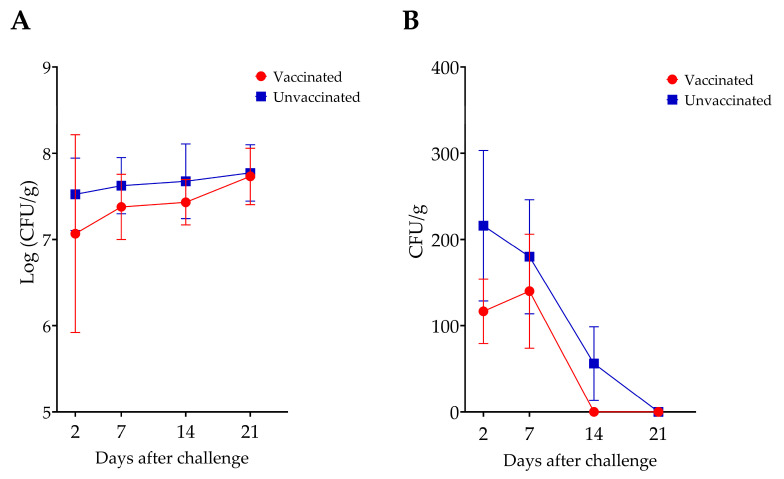
Comparison of *S*. Enteritidis counts in the cecum (**A**) and spleen (**B**) of vaccinated and unvaccinated chickens. There was no statistically significant difference in *Salmonella* counts between the two groups.

In Experiment I, a transient reduction in *Salmonella* counts was observed in the cecum and spleen of the vaccinated group, compared to the unvaccinated group, which lasted for two weeks (Figure 4). However, these reductions were not statistically significant. Additionally, no differences in bacterial counts were observed in the liver between groups.

### 3.3. Transcriptomic Analysis of Chicken Immune Response

**Figure 5:**
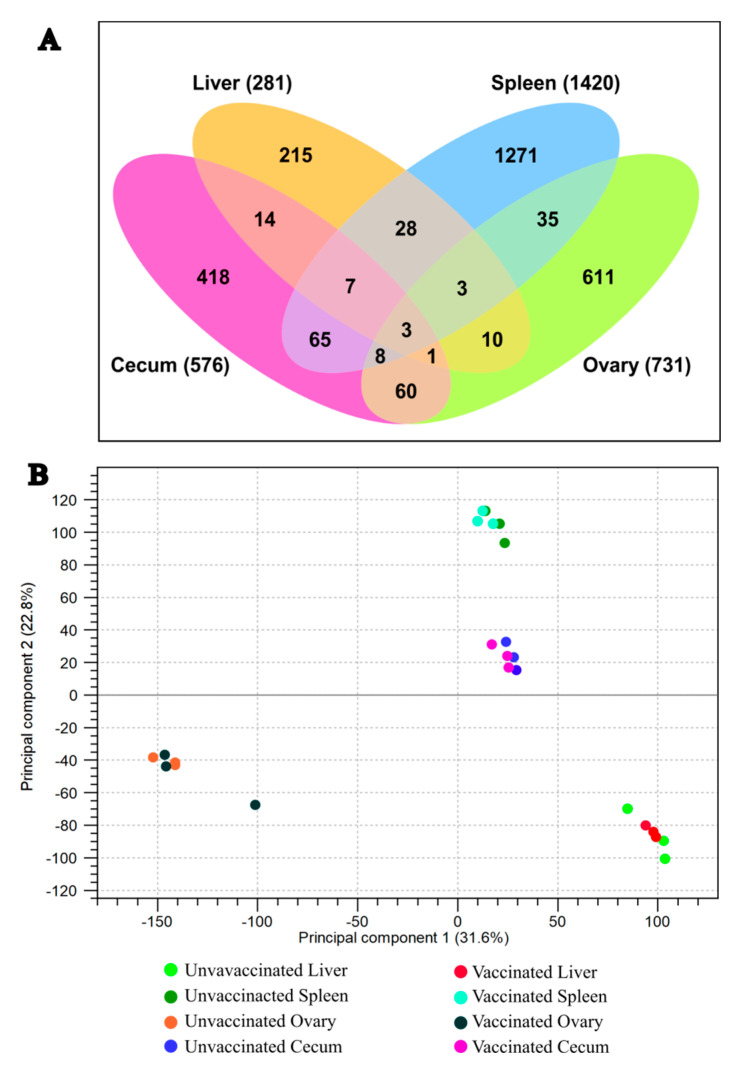
(**A**) Venn diagram illustrating differentially expressed genes in each organ. (**B**) PCA plot showing sample clustering by organ.

NovaSeq sequencing yielded an average sequence coverage of more than 50 million reads per sample. This accounted for total counts of 281, 1420, 731, and 576 genes in the liver, spleen, ovary, and cecum, respectively (Figure 5). Following filtering based on a false discovery rate (FDR ≤ 0.05), (FC ≥ 2.0), and *p*-value (≤0.01), differential expression analysis identified 252 genes in the liver (80 upregulated, 172 downregulated), 844 in the spleen (130 upregulated, 714 downregulated), 731 in the ovary (670 upregulated, 61 downregulated), and 333 in the cecum (128 upregulated, 205 downregulated) (Figure 5A).

**Figure 6:**
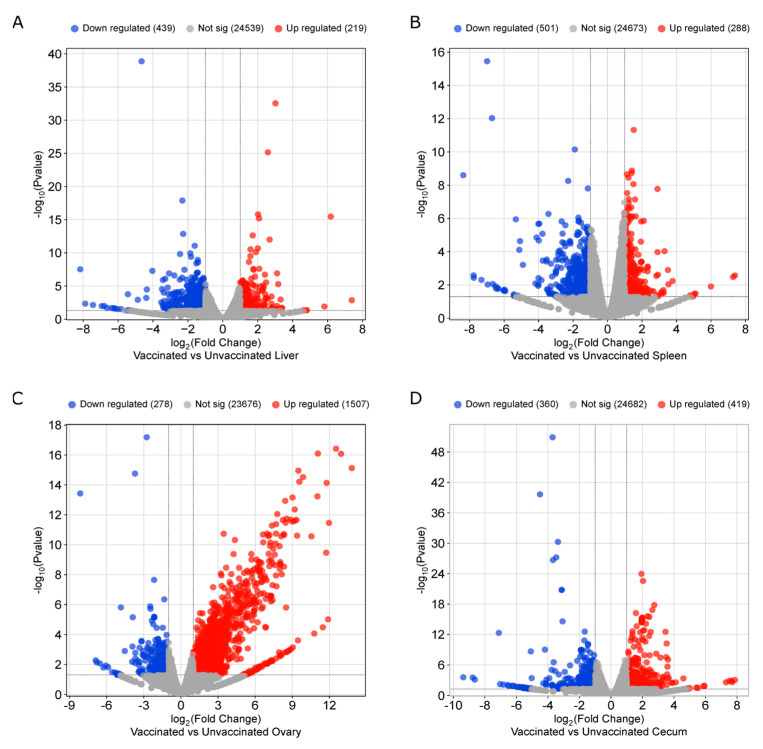
Volcano plots depicting differential gene expressions in (**A**) Liver, (**B**) Spleen, (**C**) Ovary, and (**D**) Cecum. (FDR ≤ 0.05, FC ≥ 2.0, and *p*-value ≤ 0.01).

Principal component analysis (PCA) was conducted to assess the heterogeneity among replicates and between treatments. The PCA plot showed clear clustering of groups, indicating consistent transcriptomic changes in biological replicates of the vaccinated and unvaccinated groups (Figure 5B). Differential expression of genes in each tissue (liver, spleen, ovary, and ceca) is presented in Figure 6A–D, respectively.

#### 3.3.1. Enrichment Analysis

A Gene Ontology (GO) enrichment analysis was performed on differentially expressed genes to identify the important immune pathways activated in response to vaccination. Immune-related genes to be analyzed were identified by a literature survey [31,32,33] and by searching for the keyword “immune” in the gene database that fulfilled inclusion criteria (FDR ≤ 0.05, FC ≥ 2.0, and *p*-value ≤ 0.01). A total of 89 immune-related genes were differentially expressed in all tissues.

#### 3.3.2. Immune Response in Tissues

**Ovary:** A total of 22 immune-related genes were differentially expressed in ovarian tissues, of which 21 showed an upregulation and one showed downregulation (Supplementary Document S1—Ovary). Ovarian tissues showed the largest fold changes in the differential expression of immune-related genes. In particular, antimicrobial peptides such as AvBD10 (avian beta-defensin 10), CATH3 (cathelicidin-3), and GNLY (granulysin), which exert direct antibacterial activity and play key roles in immune regulation, were significantly upregulated. This pattern suggests strong innate immunity, which is crucial for controlling of *Salmonella* infection [34,35,36].

In addition to antimicrobial peptides, the chickens in the vaccinated group exhibited an upregulation of genes that are involved in bacterial detection and defense-related immune modulation. For example, LGALS3 (Galectin-3) and PGLYRP2 (peptidoglycan recognition protein 2), which function as pattern recognition receptors for pathogen- and damage-associated molecular patterns, were highly upregulated in the vaccinated chickens [37,38]. In contrast, CD163L (CD163-like molecule), which functions as an immune sensor [39] for pathogens, was downregulated. Furthermore, genes such as MASP1 (mannose-associated serine protease 1) SPP1, RARRES2 (retinoic acid receptor responder 2), PTPN6 (protein tyrosine phosphatase non-receptor type 6), and TIMP2 (tissue inhibitor of metalloproteinases 2), which are involved in inflammatory and innate immune responses, and LOC100859272 in humoral immunity against bacteria were upregulated, showing an overall stimulation of the chicken immune system.

**Spleen:** The spleen showed the greatest number (*n* = 40) of immune-related DGEs, compared to other tissues (Supplementary Document S2—Spleen). Genes related to both innate and adaptive immune stimulation were observed, of which 13 were upregulated and 27 were downregulated. Key upregulated genes of innate immune functions included TLR5 (Toll-like receptor 5), which is a pattern recognition receptor that specifically detects bacterial flagellin [40] and LY96 (lymphocyte antigen 96), which serves as an essential co-receptor for TLR4-based LPS detection and activation of TLR4-mediated signaling pathways [41]. These Toll-like receptor pathway components facilitate the spleen’s role in filtering bacterial pathogens from the circulation [40] and trigger downstream immune cascades, including cytokine release, antimicrobial peptide production, and recruitment of effector cells [42]. It was also observed that CCR6 (C-C chemokine receptor 6), which mediates the recruitment of CD4+ T cells, B cells, and dendritic cells to mucosal sites and, thereby, contributes adaptive immune priming at mucosal surfaces, was upregulated [43,44,45]. Another interesting finding was the upregulation of EXFABP (Extracellular fatty acid-binding protein), which can bind with bacterial ferric siderophores and has been identified to possess antibacterial properties [46,47,48]. Furthermore, CACTIN (cactin, spliceosome C complex subunit), which is involved in the negative regulation of interferon and Toll-like receptor signaling pathways, was downregulated, indicating positive immune stimulation. However, IRF5 (Interferon Regulatory Factor 5), which is involved in the modulation of innate immunity and activation and M1 polarization of macrophages, was also downregulated [49,50].

Certain genes associated with adaptive immune responses also exhibited differential expression. For example, the BTK (Bruton’s tyrosine kinase) gene, which is involved in the stimulation and regulation of B lymphocytes, was downregulated. This may reflect negative feedback regulation in already stimulated B cells engaged in antibody production. Similarly, transcriptional factors, such as ANXA1 (Annexin A1), STAT6 (signal transducer and activator of transcription-6) [51], and SASH3 (SAM And SH3 Domain Containing 3) [52], which are involved in T cell-mediated immune responses, were also downregulated.

**Cecal wall:** Transcriptomic analysis of cecal tissues identified 17 immune-related genes, of which 10 were upregulated and 7 were downregulated (Supplementary Document S3—Cecum). CD36, which functions as a pattern-recognition receptor, was significantly upregulated in vaccinated chickens. This receptor functions in conjunction with Toll-like receptors to facilitate bacterial detection and phagocytosis at the mucosal surfaces [53,54,55]. CCLI8 (C-C chemokine ligand 18), which is involved in chemotactic activity and immune modulation [56], also showed robust upregulation. Additionally, RASD1, MAFF, and ATF3, genes associated with immunoregulation, were significantly upregulated in the vaccinated group [57]. MMP1 (matrix metalloproteinase 1), which plays a critical role in maintaining mucosal integrity [58], was upregulated in vaccinated chickens, consistent with previous observations during *Salmonella* infection studies in chickens [59]. CCL17 (C-C motif chemokine ligand 17), a key mediator of innate immunity, was also upregulated. This chemokine promotes macrophage polarization and dendritic cell development, thereby enhancing antigen presentation capacity [60,61]. Conversely, BCL11B, which is involved in T cell development and differentiation [62], and immunomodulatory genes, such as TNFSF10 (TNF Superfamily Member 10) and NFKBIZ [63], were downregulated.

**Liver:** A total of 10 immune-related genes were differentially expressed in the liver, with 5 upregulated and 5 downregulated (Supplementary Document S4—Liver). Most of these genes were not directly related to host immunity against bacterial pathogens. However, genes such as TNFSF10 (TNF superfamily member 10), NFIL3 (nuclear factor, interleukin 3 regulated), and NPAS2 (neuronal PAS domain protein 2), which are involved in innate immune regulation, were significantly upregulated.

### 3.4. Experiment II—Efficacy of Maternally Acquired Anti-InvG Antibodies in Protecting Progeny Chicks Against Salmonella Challenge

**Figure 7:**
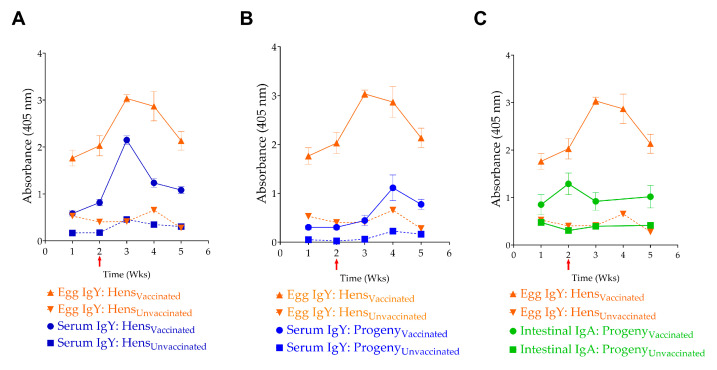
Transfer of maternal antibodies. Correlation of (**A**) egg IgY levels with serum IgY levels in hens, (**B**) egg yolk IgY and serum IgY levels in progeny chicks, and (**C**) egg IgY and intestinal sIgA levels in progeny. The red Arrow indicates the 3rd vaccination event, and the time indicates weekly egg collection events.

Vaccinated hens and their progeny chicks demonstrated significantly higher levels of anti-InvG serum IgY antibodies, compared to the unvaccinated cohort, with a significant spike in serum IgY after the third booster vaccination (Figure 7A). However, the elevated serum IgY titers declined rapidly, as evidenced by measurements taken 2 weeks after the third vaccination, which corresponded to the fifth week of egg collection. This transient response observed can be attributed to the characteristics of InvG as a subunit vaccine and its parenteral route of administration. Despite three intramuscular administrations spaced two weeks apart, the vaccine resulted in no adverse reactions in the chickens during both experiments, with no evidence of apparent illness, body weight changes, or lesions at the injection site.

#### 3.4.1. Transfer of Anti-InvG Antibodies to Progeny

IgY antibodies produced in adult hens were efficiently transferred to their progeny via the egg yolk. This maternal antibody transfer was demonstrated by the correlation between serum IgY and intestinal sIgA levels in progeny of vaccinated hens and the corresponding serum IgY and egg yolk IgY levels in vaccinated hens (Figure 7). For example, the characteristic spike in serum IgY levels observed in adult hens, following the third vaccination, was reflected in both egg yolk IgY and serum IgY and sIgA levels in progeny chicks. Notably, although vaccinated adult hens did not show a statistically significant increase in sIgA levels in their intestinal washings, the progeny of vaccinated hens demonstrated significantly higher sIgA levels than the control group.

#### 3.4.2. Reduction of *Salmonella* Colonization

**Figure 8:**
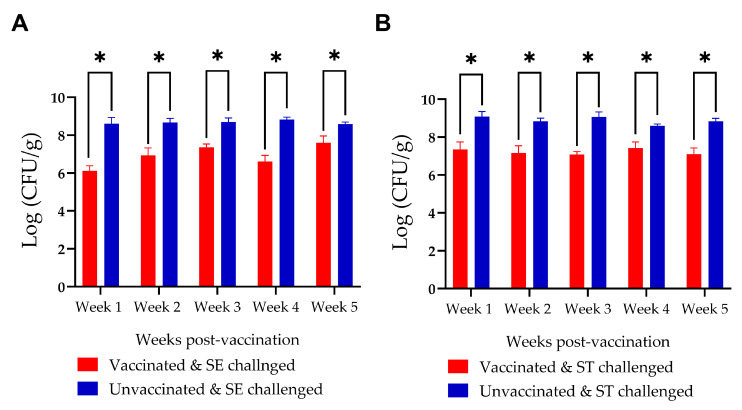
Comparison of *Salmonella* counts in the ceca of progeny chickens from vaccinated and unvaccinated hens following challenge with (**A**) *Salmonella* Enteritidis (SE) and (**B**) *Salmonella* Typhimurium (ST). Progeny from vaccinated hens exhibited a significant reduction in *Salmonella* counts in both the SE- and ST-challenged groups. * denotes a statistically significant difference between groups.

In Experiment II, significant levels (up to 1 log unit) of reduction in cecal *Salmonella* counts were observed in the progeny of vaccinated hens with both *S*. Enteritidis and *S*. Typhimurium challenge (Figure 8). The *S*. Kentucky challenge group was excluded from the analysis because of bacterial overgrowth on enumeration plates, even at the highest dilution, which prevented accurate enumeration of *Salmonella*. This bacterial overgrowth was observed exclusively in the *S*. Kentucky-challenged groups.

### 3.5. Experiment III—Persistence of Maternal Anti-InvG Antibodies in Serum and Egg Yolk

**Figure 9:**
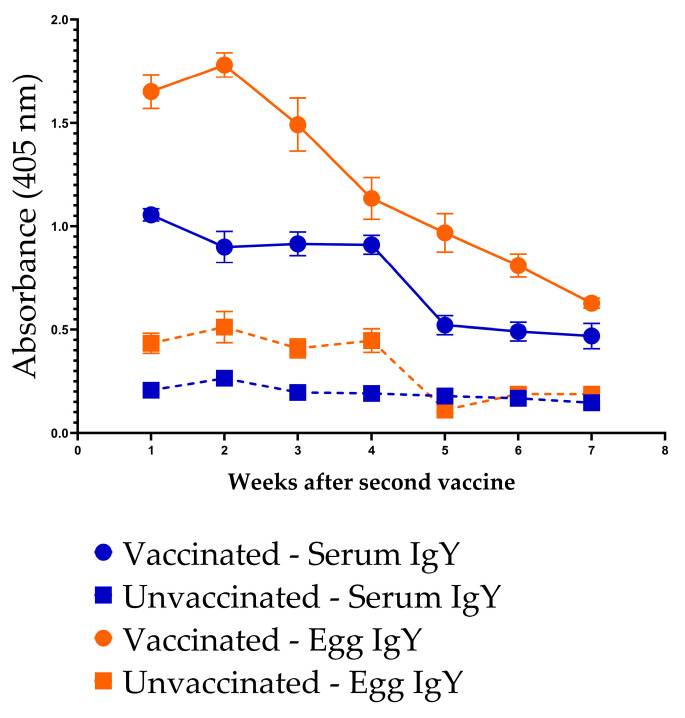
Persistence of anti-InvG IgY antibodies in the serum and egg yolk of vaccinated and unvaccinated chickens.

Following the second dose of InvG, vaccinated hens exhibited robust anti-InvG IgY responses in both serum and egg yolk. Serum IgY levels remained elevated and relatively stable through four weeks post-vaccination, followed by a gradual decline over the subsequent weeks, while remaining consistently higher than in unvaccinated controls throughout the study period (i.e., up to 7 weeks post-vaccination). Egg yolk IgY levels in vaccinated hens peaked during the first two weeks, after the second dose, and declined steadily thereafter, while remaining elevated for up to seven weeks. In contrast, both serum and egg yolk IgY levels in unvaccinated hens remained low and unchanged across all time points. These data demonstrate sustained systemic antibody responses following two doses of InvG vaccine and efficient transfer of vaccine-induced IgY into the egg yolk over time (Figure 9).

## 4. Discussion

This study highlights the potential of InvG as a novel vaccine candidate against NTS in poultry. Our findings indicate that InvG, a conserved component of the T3SS of all *Salmonella* serovars, induces strong antibody responses in vaccinated hens, their eggs, and progeny, and reduces *Salmonella* colonization in the progeny. Its high degree of conservation across *Salmonella* serovars makes InvG an attractive target for vaccines that could protect against multiple serovars, thereby overcoming the limitations of current serovar-specific vaccines.

Current vaccination strategies for controlling *Salmonella* in commercial layer chickens predominantly involve the use of live attenuated, inactivated, and subunit vaccines. Even though both live and killed vaccine types have demonstrated significant efficacy in reducing *Salmonella* colonization and contamination within poultry flocks, the protection is superior with live vaccines [64]. *S*. Enteritidis killed and *S*. Typhimurium live attenuated vaccines, alone or in combination with vaccines against other infectious diseases, are used extensively by the poultry industry. Current standard vaccination regimens involve vaccination with a live attenuated *S*. Typhimurium vaccine by spray at one-day-old, followed by boosters at 2–3 weeks and optionally at 6–10 weeks, with a final killed *S*. Enteritidis vaccine administration at 12–14 weeks, before laying [64]. Notably, vaccination during the laying period has been uncommon in the United States, though Europe has authorized Primun *Salmonella* E for use during laying, representing a significant advancement [65].

However, the serovar specificity of these vaccines creates a competitive advantage for serovars that are not targeted by the vaccine. Likely, as a consequence, serovars such as Heidelberg and Kentucky have recently emerged as predominant serovars in poultry production [66]. Concurrently with their widespread prevalence in poultry as an intestinal colonizer, these serovars have gained multidrug resistance, further exacerbating their public health impact. For example, the emergence of multidrug-resistant *S*. Infantis has become particularly problematic, with this serovar now representing a persistent epidemic strain in poultry [67]. Another key challenge is the variability of field performance, with vaccine efficacy varying considerably under different environmental conditions and management practices [68]. The inability to achieve complete *Salmonella* elimination remains a fundamental issue, since vaccinated chickens can still harbor and intermittently shed *Salmonella* under certain conditions, such as the onset of lay and other environmental stressors.

While vaccination is not federally mandated in the United States, it is strongly encouraged to vaccinate commercial layer chickens under the FDA’s Egg Safety Rule, which requires comprehensive measures to prevent *S.* Enteritidis contamination in shell eggs [69]. A significant regulatory breakthrough occurred in April 2024 when the USDA-FSIS announced that vaccine-derived *Salmonella* strains would be excluded from performance categorization [70]. This policy change removes a major barrier to vaccine implementation by preventing establishments from being penalized when vaccine strains are occasionally detected in raw poultry products during routine sampling.

Subunit vaccines, particularly those utilizing OMPs, offer a promising strategy for controlling *Salmonella* infections. In contrast to conventional whole-cell vaccines, subunit vaccines are designed to elicit targeted immune responses against specific antigenic components. These subunit vaccine platforms offer enhanced biosafety profiles, compared to live-attenuated or inactivated whole-cell preparations, which present risks including potential reversion to virulence [71]. Multiple OMPs have been tested previously [72,73,74] and are considered highly effective antigens and immune stimulants, since their surface exposure on bacteria makes them readily recognizable by the host immune system [75]. A significant factor in their effectiveness against multiple serovars is the conserved nature of these protein antigens, which provide cross-protection against various *Salmonella* serovars [76].

Our investigation focused primarily on IgY levels as the primary indicator of immune response, given that IgY is the predominant antibody in chicken serum and egg yolk, while IgM and IgA are present in minimal concentrations in serum [77]. This approach was sufficient to evaluate both immunogenicity and passive transfer of immunity to the progeny. Prior to immunization, all chickens were screened and confirmed negative for *Salmonella* by cloacal swabs. Chicks for Experiments I and III originated from SPF flocks, while Experiment II utilized chicks from a commercial hatchery without *Salmonella* vaccination, as verified by hatchery records. This controlled baseline ensured that humoral immune responses detected in vaccinated chickens reflect InvG-specific immunization, rather than prior *Salmonella* exposure. It must be noted that only around 30% of IgY in a hen’s plasma is transferred to eggs, whereas only 1% of IgA is transferred from a hen’s plasma to eggs [78]. However, ELISA OD values for anti-InvG antibodies cannot be directly compared between egg yolk and serum samples, due to matrix interference. The higher protein and lipid content of egg yolk results in elevated background absorbance, artificially amplifying OD readings. Consistent with previous research, we also observed a strong correlation between egg yolk and serum antibody levels in both hens and progeny [78,79]. Most significantly, the vaccinated progeny showed substantial reductions in intestinal colonization by heterologous *Salmonella* serovars, highlighting the potential of InvG to protect against heterologous serovars.

In Experiment II, a sharp spike of antibodies against InvG was observed after the third dose of the vaccine. This spike was followed by a steep reduction in antibody levels in both the serum and egg yolk of vaccinated hens. However, the persistence of anti-InvG IgY could not be established in this study because the study was continued only for a period of 3 weeks after the third vaccine dose in hens. Subsequently, Experiment III was conducted to understand the persistence of these antibodies, and it was evident that anti-InvG IgY could last at significantly higher levels in the vaccinated hens, compared to unvaccinated hens.

While subunit vaccines typically do not elicit robust mucosal immune responses in vaccinated chickens, our findings revealed significant levels of sIgA in the intestinal washings of the progeny and marginally significant levels in the adult hens. This finding is significant because the mucosal sIgA response is the first line of defense against enteric pathogens. The sIgA response may be attributed to the maturity of the hen’s immune system, compared to that of young chickens, as mature immune systems are known to generate a robust immune response, including enhanced sIgA production [80]. This observation aligns with previous reports of successful mucosal immune responses induced by subunit vaccines, particularly in studies targeting the major FliC antigenic site of *S.* Enteritidis [81] and those utilizing oligopeptides combined with anti-chicken CD40 monoclonal antibodies for targeting antigen-presenting cells [82]. Furthermore, it has been observed that adjuvants like QuilA could lead to a balanced Th1 and Th2 response, leading to both effective humoral and cellular responses, providing better mucosal immunity [83]. In chickens, sIgA can be transferred from the hen to the progeny through the egg, providing an important layer of passive mucosal immunity during the early life of chicks [21,78]. This condition aligns with our observation of robust IgA responses in the intestinal washings of progeny chickens from vaccinated hens. Passively transferred sIgA plays an important role in defense against pathogens at mucosal surfaces, including intestinal colonization of *Salmonella* [84,85].

Our previous observations indicated that intramuscular vaccination with InvG fails to induce a strong, sustained IgA response (unpublished data). However, in Experiment I, during the first week after the *Salmonella* challenge, significantly elevated levels of intestinal IgA were detected in the vaccinated-challenged group, compared to the unvaccinated-challenged group, although this increase was transient. By one-week post-challenge, the sIgA titers in the vaccinated group had declined below those observed in the unvaccinated-challenged group. Over time, the intestinal sIgA levels in the unvaccinated group continued to increase throughout the experiment (Figure 3B). Similar observations have been made in previous studies examining mucosal immune responses, where sham-vaccinated chickens developed higher levels of secretory IgA against the antigen, compared to the unvaccinated group after challenge [71,86]. This observation suggests that the higher *Salmonella* counts in the unvaccinated group might have stimulated the immune system, triggering an increase in IgA synthesis.

In addition to the efficacy of InvG as a vaccine, demonstrated by antibody response, transcriptomic analysis showed a clear stimulation of both innate and adaptive immune responses in vaccinated chickens. However, because samples for RNA extraction were collected 2 weeks after primary vaccination and 1 week after the booster, it is likely that much of the early innate immune response, which is typically most prominent shortly after antigen exposure, had waned by the time of sample collection for the transcriptomics analysis. It should also be noted that some of the genes related to innate immunity were adopted from mammalian databases, as many immune pathway genes are shared among animal classes.

In the ovary, several important innate immune genes, including antimicrobial peptides, such as AvBD10, CATH3, and GNLY, and pattern recognition receptors, such as LGALS3 and PGLYRP2, were significantly upregulated, suggesting enhanced pattern recognition and frontline antibacterial activity [34,35,36,87,88]. In contrast, CD163L, a macrophage scavenger receptor, was downregulated, which may indicate regulatory modulation of macrophage activity in the ovarian environment [39,89]. Additional upregulation of genes, such as MASP1, SPP1, RARRES2, PTPN6, and TIMP2, suggests overall activation of innate immune pathways, including complement activation, cytokine regulation, and tissue remodeling processes [90,91,92,93,94,95] that could contribute to protection against NTS colonization in the ovaries. The improved immune defense mechanisms observed in the ovary in response to vaccination indicate that InvG is effective at reducing transovarian (vertical) transmission of *Salmonella* to eggs, thereby decreasing egg-associated foodborne salmonellosis and interrupting the transmission cycle from infected hens to their progeny.

The spleen transcriptome revealed the upregulation of pattern recognition receptor genes TLR5 and LY96, indicating enhanced detection of bacterial pathogens and downstream signaling [40,41]. Elevated CCR6 and EXFABP expression suggests the recruitment of immune cells to mucosal sites and direct antibacterial activity, respectively [43,44,45,46]. The downregulation of CACTIN, a negative regulator of the interferon and TLR pathways, further supports active immune stimulation. However, decreased expression of IRF5, BTK, ANXA1, STAT6, and SASH3 likely represents compensatory feedback mechanisms in activated immune cells rather than immunosuppression, as evidenced by concurrent increase in serum antibody titers in vaccinated chickens.

In cecal tissues, CD36 upregulation enhances bacterial detection and phagocytosis at the mucosal surfaces. The concurrent upregulation of chemokines CCLI8 and CCL17 suggests enhanced recruitment and activation of immune cells, with CCL17 specifically promoting macrophage polarization and dendritic cell maturation, thereby strengthening antigen presentation capacity [56,60,61]. This enhanced antigen presentation is crucial for establishing both innate and adaptive immunity against *Salmonella*. The downregulation of immunomodulatory genes (BCL11B, TNFSF10, NFKBIZ) suggests a balanced immune regulation. Limited immune responses were observed in hepatic tissues, potentially reflecting the liver’s unique tolerogenic microenvironment, which develops due to constant exposure to a diverse array of dietary antigens and commensal bacteria-derived products via the portal blood supply [96].

The control of *Salmonella* in poultry production systems heavily relies on economically viable and operationally practical vaccination strategies [97]. As the industry faces challenges from multiple *Salmonella* serovars, the development of vaccines offering broader cross-protection is essential for maintaining production efficiency and meeting food safety requirements. A prospective avenue for further improvement of InvG as an effective poultry *Salmonella* vaccine involves the delivery of InvG as a live vector vaccine using a suitable vector organism, facilitating its administration via the mucosal route. Since live vaccines can be administered through vaccination routes, such as spraying or drinking water, they are particularly important in meeting industry requirements of cost-effectiveness and ease of administration [13,98]. Furthermore, they provide the added advantage of inducing a mucosal immune response locally in the exposed mucosa, unlike routes such as intramuscular vaccination. Live *Salmonella* vaccines typically demonstrate superior immunogenicity, compared to killed or subunit vaccines, as they stimulate both humoral and cell-mediated immune responses [13,99]. Additionally, they provide supplementary benefits to one-day-old chicks by establishing beneficial gut flora, potentially facilitating pathogen exclusion through competitive inhibition. However, it should also be noted that sterilizing immunity is hardly achieved against NTS in poultry, even with many available vaccines [64]. Since anti-InvG antibodies lead to significant reductions in intestinal colonization of heterologous *Salmonella* serovars, it can be speculated that this antigen would be a good candidate for a future poultry *Salmonella* vaccine. However, due to methodological limitations with *S*. Kentucky enumeration and animal housing facility constraints, InvG was evaluated against only two serovars. Further research incorporating additional serovars such as *S*. Heidelberg and *S*. Infantis, which have become increasingly prevalent in poultry flocks, is required to corroborate the efficacy of InvG against additional serovars.

## 5. Conclusions

This study evaluated the potential of InvG, a highly conserved structural component of the *Salmonella* type III secretion system, as a vaccine candidate in chickens. Although intramuscular vaccination with InvG did not provide adequate protection against *Salmonella* colonization, it elicited strong systemic antibody responses in hens, allowed efficient maternal transfer of IgY and mucosal IgA to progeny, and reduced intestinal colonization by heterologous *Salmonella* serovars in newly hatched chicks. Transcriptomic analyses further revealed coordinated activation of innate and adaptive immune pathways across multiple tissues, supporting the biological relevance of the observed protective effects. Although protection in adult hens was modest, the consistent immunogenicity, cross-serovar potential, and benefits conferred through maternal immunity in progeny chicks highlight the suitability of InvG as a vaccine candidate for *Salmonella* control. Future studies focusing on alternative delivery platforms, including live-vector or mucosal vaccination strategies, will improve the translational potential of InvG for *Salmonella* control in commercial layer production systems.

## Data Availability

The original contributions presented in this study are included in the article/Supplementary Material. Further inquiries can be directed to the corresponding author.

## Supplementary Materials

The following supporting information can be downloaded at: https://www.mdpi.com/article/10.3390/vaccines14010068/s1, Supplementary Document S1—Ovary; Supplementary Document S2—Spleen; Supplementary Document S3—Cecum; Supplementary Document S4—Liver.

## Abbreviations

The following abbreviations are used in this manuscript:

NTS	Non-typhoidal *Salmonella*
ELISA	Enzyme Linked Immunosorbent Assay
BPW	Buffered Peptone Water
MDR	Multi Drug Resistance
T3SS	Type 3 Secretion System
SPF	Specific Pathogen Free
sIgA	Secretory IgA
LB	Luria-Bertani
XLT-4	Xylose Lysine Tergitol-4
PBS	Phosphate-buffered Saline
OMP	Outer Membrane Protein
PM	Postmortem
SE	*Salmonella* Enteritidis
ST	*Salmonella* Typhimurium
IACUC	Institutional Animal Care and Use Committee
TLR	Toll-Like Receptor
